# Comparison of response patterns in different survey designs: a longitudinal panel with mixed-mode and online-only design

**DOI:** 10.1186/s12982-017-0058-2

**Published:** 2017-03-21

**Authors:** Nicole Rübsamen, Manas K. Akmatov, Stefanie Castell, André Karch, Rafael T. Mikolajczyk

**Affiliations:** 1 Department of Epidemiology, Helmholtz Centre for Infection Research, Inhoffenstr. 7, 38124 Brunswick, Germany; 2 PhD Programme “Epidemiology”, Brunswick-Hanover, Germany; 3 TWINCORE, Centre for Experimental and Clinical Infection Research, Feodor-Lynen-Str. 7, 30625 Hanover, Germany; 4 Hannover Medical School, Hanover, Germany

**Keywords:** Bias, Health survey, Internet, Longitudinal study, Mixed-mode, Online, Panel study, Participation, Response

## Abstract

**Background:**

Increasing availability of the Internet allows using only online data collection for more epidemiological studies. We compare response patterns in a population-based health survey using two survey designs: mixed-mode (choice between paper-and-pencil and online questionnaires) and online-only design (without choice).

**Methods:**

We used data from a longitudinal panel, the Hygiene and Behaviour Infectious Diseases Study (HaBIDS), conducted in 2014/2015 in four regions in Lower Saxony, Germany. Individuals were recruited using address-based probability sampling. In two regions, individuals could choose between paper-and-pencil and online questionnaires. In the other two regions, individuals were offered online-only participation. We compared sociodemographic characteristics of respondents who filled in all panel questionnaires between the mixed-mode group (n = 1110) and the online-only group (n = 482). Using 134 items, we performed multinomial logistic regression to compare responses between survey designs in terms of type (missing, “do not know” or valid response) and ordinal regression to compare responses in terms of content. We applied the false discovery rates (FDR) to control for multiple testing and investigated effects of adjusting for sociodemographic characteristic. For validation of the differential response patterns between mixed-mode and online-only, we compared the response patterns between paper and online mode among the respondents in the mixed-mode group in one region (n = 786).

**Results:**

Respondents in the online-only group were older than those in the mixed-mode group, but both groups did not differ regarding sex or education. Type of response did not differ between the online-only and the mixed-mode group. Survey design was associated with different content of response in 18 of the 134 investigated items; which decreased to 11 after adjusting for sociodemographic variables. In the validation within the mixed-mode, only two of those were among the 11 significantly different items. The probability of observing by chance the same two or more significant differences in this setting was 22%.

**Conclusions:**

We found similar response patterns in both survey designs with only few items being answered differently, likely attributable to chance. Our study supports the equivalence of the compared survey designs and suggests that, in the studied setting, using online-only design does not cause strong distortion of the results.

**Electronic supplementary material:**

The online version of this article (doi:10.1186/s12982-017-0058-2) contains supplementary material, which is available to authorized users.

## Background

The increased availability of the Internet stimulates the establishment of epidemiological studies relying solely on online data collection [1]. Recruitment for online studies can rely on either probability or convenience sampling, whereby the latter has the advantage of being able to access large sample sizes [2], but the disadvantage of limited generalizability: convenience samples recruited via the Internet have been shown to be less representative of the population that they were drawn from [2–4]. Random (probability) sampling offers a way to decrease selection bias and to increase generalisability because each individual in the sampling frame has a predefined chance of being sampled [4].

Relying solely on online data collection has obvious advantages with respect to costs and effort [5, 6], but carries the risk of low participation and a biased selection of study participants [7]. Previous research indicated that participation can be increased by using mixed-mode surveys compared to online-only surveys [5, 8]. Several studies investigated how responses differed between online and non-online responders within mixed-mode surveys [9–12]. They showed that preferred survey mode was linked to sociodemographic factors; once adjusted for those baseline differences, the majority of differential responses disappeared [11, 12]. However, these comparisons do not encompass the situation where only one mode of data collection is offered, and those willing to participate are forced to use it. Only few studies compared such single-mode studies with mixed-mode studies regarding response patterns. They compared mixed-mode (paper and online) with single-mode studies (computer-assisted, face-to-face or telephone interviews) [13, 14]. Up to now, no study included comparisons of response patterns in online data collection with response patterns in a mixed-mode survey. Also, most published studies considered only a single thematic focus. A further limitation of previous studies is the lack of adjustment for multiple testing, potentially overestimating the true differences between data collection modes.

### Study aim and research questions

We took advantage of a large population-based, longitudinal panel covering a range of epidemiological research questions. Our main study aim was to compare response patterns between an online-only and a mixed-mode survey design. To achieve this aim, we proceeded in several steps.

First, we investigated if applying online-only data collection leads to selection of participants, i.e. if the composition of participants in online-only data collection differs from that in a mixed-mode study offering choice between online and paper-and-pencil questionnaires. To explore this issue, we investigated the following questions:

1. Do response rates differ between online-only survey and mixed-mode survey (overall and stratified by age)?
2. Do sociodemographic characteristics differ between participants of the online-only survey and the mixed-mode survey?

We also looked at sociodemographic characteristics within the mixed-mode group because we hypothesised that differences between groups of participants would be more pronounced between modes of data collection within the mixed-mode group than between the survey designs. Thus, we investigated the question:

3. Do sociodemographic characteristics differ in those participants who had the choice between modes of data collection?

Lower costs for survey administration are often named as major advantages of online-only surveys [5, 6]. This advantage can, however, be outweighed by lower response rates in online-only surveys [15]. To assess cost-effectiveness in our study setting, we investigated the question:

4. Are costs per survey participant higher in a mixed-mode than in an online-only survey?

Having investigated the mentioned research questions, we finally compared response patterns between the online-only and the mixed-mode survey design by investigating the questions:

5. Do responses of participants in the online-only survey differ from those in the mixed-mode survey with respect to type of response (i.e. missing, “do not know”, or valid response)?
6. Within the valid responses, does choice of answer categories (i.e. content of response) differ between the online-only survey and the mixed-mode?
7. Can differences in response patterns be explained by selection of participants with respect to sociodemographic characteristics (if any found in step 2)?

To validate differences found in response patterns (if any), we investigated if these differences were specific to the online mode of participation. If the differences were specific to the online mode of participation, it could be expected that the differences would be even larger if respondents could choose their mode of participation.

8. Do variables, for that differences between mixed-mode and online-only mode were identified, also display differences in a comparison between online and paper-and-pencil respondents within the mixed-mode group?

## Methods

### Study design and recruitment

This analysis is based on the Hygiene and Behaviour Infectious Diseases Study (HaBIDS) designed to assess knowledge, attitudes, and practice related to infectious diseases [16, 17]. HaBIDS is a longitudinal panel with population-based sampling in four regions of the federal state Lower Saxony in Germany. We included female and male individuals aged between 15 and 69 years. Potential participants were drawn by means of proportional stratified random sampling from the respective population registries. Individuals were excluded if their primary residence was not within one of the four regions.

In January 2014, we sent invitation letters to 16,895 individuals drawn from the registries of the district of Brunswick (city with more than 10,000 inhabitants), from two nearby villages (Wendeburg and Bortfeld with less than 10,000 inhabitants each), and from the district of Vechta (six cities with less than 10,000 inhabitants each). The invitation letter included information about the study and an informed consent form. Individuals were told in the information that they could choose between participation via online or paper-and-pencil questionnaires. If an individual decided to participate, then he or she could either write an email address on the informed consent form where we should send the online questionnaires or a postal address where we should send the paper-and-pencil questionnaires. Every individual who consented to participate is called “participant” in this text.

In April 2014, we sent invitation letters to 10,000 individuals drawn from the registries of the districts of Salzgitter and Wolfenbüttel (cities with more than 10,000 inhabitants each). The invitation letter included information about the study and an informed consent form. Individuals were told in the information that the study would be conducted using online questionnaires. Participants should write an email address on the informed consent form where we should send the online questionnaires.

Participants were assigned a unique identifier (ID). All study data were stored using this ID, without any connection to personal data of the participants. Personal data (postal and e-mail addresses) were stored separately on a secure local server without Internet connection. The access was restricted to study personnel only.

### Questionnaire development and survey administration

We developed questions for the study using already published questionnaires, where applicable [18–22], and added items based on expert opinion where no validated questionnaires were available. The questions covered general health and sociodemographic characteristics, frequency of infections and infection-associated symptoms in the last 12 months, prevention measures against respiratory infections, perceptions related to adult vaccinations, to tick-borne infections, and to antibiotics. The sections considering prevention measures against respiratory infections, vaccinations, tick-borne infections, and antibiotics were designed as knowledge-attitude-practice (KAP) surveys [23].

We implemented the paper-and-pencil questionnaires using the software TeleForm Designer^®^ [24], and the online questionnaires using the software Limesurvey^®^ [25], with a design adapted to mobile phones. No item was set to mandatory in the online mode to enable comparing the proportion of missing values between modes.

Online participants received seven questionnaires between March 2014 and July 2015. The links to the online questionnaires were distributed via email, along with unique tokens for individual logins to the survey. The token was valid until a participant used the submit button on the last survey page. In case of multiple entries from a participant, we kept only the last entry for analysis. We sent a single reminder (email) to participants who had not filled in the online questionnaire within two weeks of initial invitation. All online questionnaires remained active until October 7, 2015.

To reduce postage charges, the paper-and-pencil group received two questionnaires covering the topics of online questionnaires: the first paper-and-pencil questionnaire covered the first three online questionnaires and the second one covered the remaining four online questionnaires. The paper-and-pencil questionnaires were sent out via regular mail and could be returned using pre-paid return envelopes until October 7, 2015. We sent single reminder letters 2 months after each paper questionnaire.

### Statistical analysis

In all regression analyses, we accounted for multiple testing by applying the false discovery rate (FDR) [26]. The FDR algorithm controls the proportion of significant results that are in fact type I errors instead of controlling the probability of making a type I error (which is the aim of the Bonferroni correction) [27]. We considered *p* < 0.05 as significant. All statistical analyses were performed in Stata version 12 (StataCorp LP, College Station, TX, USA [28]).

#### Response rates

We compared response rates (Response Rate 2 according to the definition by the American Association for Public Opinion Research [29]) between the mixed-mode design and the online-only design after direct age standardisation using the population structure of Lower Saxony [30] as standard population.

#### Differences in sociodemographic characteristics between and within survey designs

Most of our further analyses focus on the comparison of p values, which are, among other factors, dependent on sample size [31]. To ensure that differences in sample size do not affect our comparisons, we restricted our dataset for analyses in two steps. Firstly, we took into account only items that were not conditional on other questions (which constituted 90% of the panel questions). All of these questions could be answered on ordinal or binary scales (Additional file 1). In total, 134 items of the questionnaires from the following areas were included: general health and sociodemographic data (2 items), frequency of infections and infection-associated symptoms in the last 12 months (8 items), prevention measures against respiratory infections (35 items), adult vaccinations (34 items), tick-borne infections (37 items), and antibiotics (18 items). Secondly, we restricted our analyses to participants who filled in all six questionnaires containing the above items. Every participant who filled in all six questionnaires is called “respondent” in this text. We compared sociodemographic characteristics of respondents and non-respondents to detect differential loss to follow-up.

We compared sociodemographic characteristics of respondents between the mixed-mode design and the online-only design using χ^2^-tests and Wilcoxon rank-sum tests. In addition, we compared sociodemographic characteristics of respondents choosing different modes of participation within the mixed-mode group.

#### Costs per survey participant

We estimated how much money we had to spend per mixed-mode respondent or online-only respondent. These figures included postage and material for invitation letters as well as staff costs for printing invitation letters, entering postal and email addresses from the informed consent forms, sending questionnaires, and entering data after the paper-and-pencil survey. They did not include administrative costs for obtaining samples from the population registries nor for implementing the questionnaires in TeleForm Designer^®^ or Limesurvey^®^.

#### Differences with respect to type of response

We performed multinomial logistic regression analysis to investigate if survey design was associated with type of response (i.e. missing, “do not know”, or valid response) for all 134 considered items. We used likelihood ratio tests to compare models with survey design included as independent variable versus empty models. In order to investigate if differences with respect to sociodemographic variables were responsible for different responses, we adjusted the models for sociodemographic characteristics.

#### Differences with respect to content of response

We investigated if the content of responses differed by survey design using ordinal regression analysis for each of the 134 items. We included survey design as an independent variable and used only valid responses (i.e. excluding missing and “do not know” responses). Prior to the analysis, we performed Brant tests of the proportional odds assumption [32]. Then, we used multivariable ordinal regression in order to adjust for differences in variables between mixed and online-only mode: age at baseline (fitted as fractional polynomial [33]), sex, and highest completed educational level.

#### Validation within the mixed-mode group

We investigated if variables, for that differences between mixed-mode and online-only mode were identified, displayed also differences in a comparison between online and paper-and-pencil respondents within the mixed-mode group. If the differences were specific to the online mode of participation, it could be expected that the differences would be even larger if respondents could choose their mode of participation. In this analysis, we only included mixed-mode respondents from the district of Brunswick to ensure homogeneity of the group. We assessed the meaning of observing differences in the same k items in both analyses based on probability of random k or more differences assuming a hypergeometric distribution.

## Results

### Response rates

**Fig. 1:**
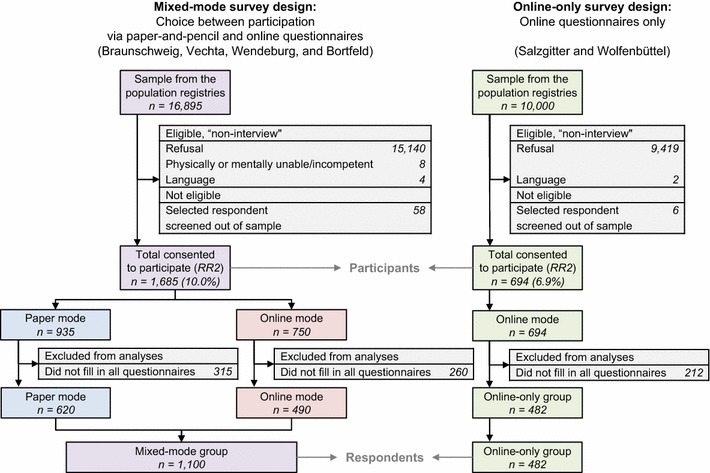
Participant flow diagram. RR2: Response Rate 2 according to the definition by the American Association for Public Opinion Research [29]

**Fig. 2:**
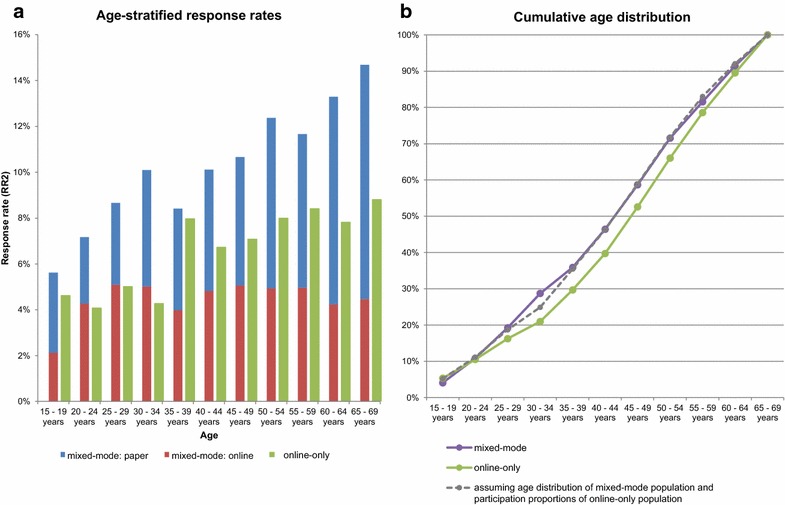
Response rates in the HaBIDS study by age group. **a** Age-stratified response rates; **b** cumulative age distribution. RR2: Response Rate 2 according to the definition by the American Association for Public Opinion Research [29]

Overall, 2379 (8.9%) of the invited individuals consented to participate in HaBIDS (Fig. 1). Response rate was higher among females than males (10.9 vs. 7.3%, respectively, *p* < 0.001). This difference was consistent in both survey designs. In both groups, the response rate was highest in the oldest age group, i.e. between 65 and 69 years, but the effect was much stronger in the mixed-mode group (Fig. 2a). There was a difference in the source population of the studied regions with respect to age (Additional file 2); we accounted for this by direct age standardisation: The crude response rate was 10.0% in the mixed-mode group (with 55.5% of the participants selecting paper questionnaires) and 6.9% in the online-only group (Fig. 1), after standardisation, the adjusted response rate was 10.3% in the mixed-mode group and 6.8% in the online-only group. The participants in the mixed-mode group were significantly younger than those in the online-only group (median age of 47 vs. 50 years, respectively, *p* = 0.009, Table 1). If the age structure in the two regions invited for online-only had been the same as in the two regions invited for mixed-mode, then the cumulative age distributions among participants would have been similar in both groups (Fig. 2b).

**Table 1:** Characteristics of the respondents in the HaBIDS study by survey design group

	Mixed-mode group [n^a^ (%)]	Online-only group [n^a^ (%)]	*p* value^b^	Within mixed-mode		
				Paper-and-pencil [n^a^ (%)]	Online [n^a^ (%)]	*p* value^c^
	N = 1110	N = 482		N = 620	N = 490	
Sex			0.71			<0.001
Female	683 (61.6)	292 (60.6)		417 (67.4)	266 (54.3)	
Male	426 (38.4)	190 (39.4)		202 (32.6)	224 (45.7)	
Age at baseline (March 2014), median (IQR)	47 (34–57)	50 (39–59)	0.009^d^	50 (36–60)	44 (32–53)	<0.001^e^
Marital status [n^a^ (%)]			0.04			0.15
Married	656 (59.4)	298 (64.6)		363 (58.7)	293 (60.3)	
Unmarried	333 (30.2)	110 (23.9)		181 (29.3)	152 (31.3)	
Other (divorced, widowed)	115 (10.4)	53 (11.5)		74 (12.0)	41 (8.4)	
Highest completed educational level			0.67			<0.001
Lower secondary education or apprenticeship	336 (30.4)	139 (30.1)		229 (36.9)	107 (22.0)	
Still at upper secondary school	25 (2.3)	10 (2.2)		21 (3.4)	4 (0.8)	
University entrance qualification (upper secondary education or vocational school)	289 (26.1)	134 (29.0)		152 (24.5)	137 (28.1)	
University degree	457 (41.3)	179 (38.7)		218 (35.2)	239 (49.1)	
Access to the Internet			0.008			<0.001
Yes	1068 (96.8)	451 (99.1)		584 (94.5)	484 (99.8)	
No	35 (3.2)	4 (0.9)		34 (5.5)	1 (0.2)	

^a^Differences to total N due to missing values, proportions excluding missing values

^b^χ^2^ test comparing mixed-mode design group in total with online-only design group (missing values were not considered)

^c^χ^2^ test comparing paper mode with online mode (missing values were not considered)

^d^Wilcoxon rank-sum test comparing mixed-mode design group in total with online-only design group

^e^Wilcoxon rank-sum test comparing paper mode with online mode

### Differences in sociodemographic characteristics between and within survey designs

Of those who consented to participate, 1592 (66.9%) participants filled in all questionnaires (“respondents”). Comparing the 1592 respondents and the 787 non-respondents, median age (48 vs. 45 years, *p* = 0.0008) and percentage of females (61.3 vs. 53.5%, *p* = 0.02) were higher among respondents, while marital status, education, and access to the Internet did not differ significantly (data not shown). There was also no evidence of differential loss to follow-up with respect to survey design or mode of data collection (data not shown).

Among the 1592 respondents, there was a significant difference regarding marital status between the mixed-mode and the online-only group; however, marital status was strongly correlated with age, and the difference disappeared after adjusting for age. Both groups did not differ significantly from each other regarding sex or level of education.

In contrast to small differences between the mixed-mode and online-only respondents, those choosing different modes within the mixed-mode design displayed stronger differences (Table 1). The median age (50 years in the paper mode vs. 44 years in the online mode, *p* < 0.001, Table 1) and the percentage of women were higher among those participating on paper (67.4 vs. 54.3% in the online mode, *p* < 0.001, Table 1). Above the age of 40 years, there was a significantly higher percentage of women in the paper mode than in the online mode (*p* < 0.001), while the percentages of women and men did not differ significantly in the age groups below 40 years (*p* = 0.23). There was also a difference with respect to education, with a lower percentage of well-educated participants among those participating on paper (35.2 vs. 49.1% in the online mode, *p* < 0.001, Table 1), independently of age.

### Costs per survey participant

We estimated that we had to spend approximately 17 Euros for one mixed-mode respondent and approximately 13 Euros for one online-only respondent. The higher costs per mixed-mode respondent were due to expenses for postage and for staff to print and post the paper questionnaires as well as for entering the data after the paper-and-pencil survey.

### Differences with respect to type of response

Among the 134 items investigated using multinomial regression, 17 items showed a significant association between survey design group and type of response after adjusting for multiple testing using FDR (Additional file 3). When adjusting for age, sex, and education, differences disappeared with only one item remaining significantly different between groups (addressing prevention of respiratory infections; Additional file 3). This difference was mainly between “do not know” responses and valid responses: the predicted probabilities for having a missing, a “do not know” or a valid response were 1.7, 27.5, and 70.8% in the mixed-mode group and 0.5, 11.0, and 88.5% in the online-only group, respectively.

### Differences with respect to content of response

**Fig. 3:**
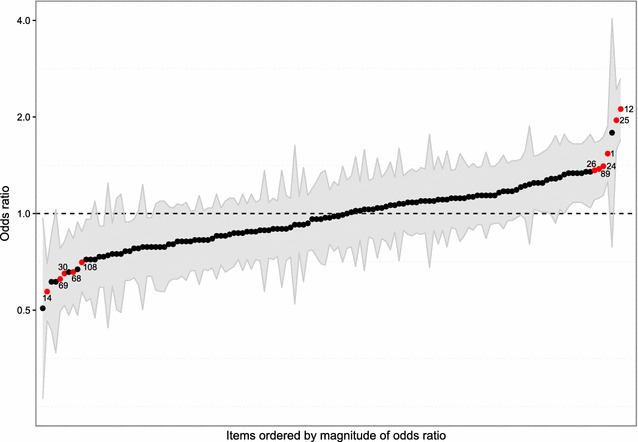
Results of the ordinal regression analyses for each of the 134 items. Ordinal regression analyses with survey design group as independent variable and content of response as dependent variable (reference: online-only), adjusted for age at baseline, sex, and highest completed educational level. *Black circle* Odds ratio of the ordinal regression analysis; *red circle* Odds ratio significantly different from one after controlling the FDR (number of item: compare Additional file 1); *grey ribbon* 95% confidence intervals of the respective odds ratio

After adjusting for multiple testing using FDR, 18 of the 134 estimates showed odds ratios (OR) significantly different from one indicating a differential response between survey design groups (Additional file 4). When adjusting for age at baseline, sex, and highest completed educational level, 11 OR were still significantly different from one (Fig. 3; Additional file 4). One of these items was related to frequency of infections, six items were related to knowledge about prevention measures, two items were related to attitudes regarding prevention measures, and two items were related to practice regarding measures for prevention against infections. For six of these items, mixed-mode respondents were less likely to select higher answer categories than online-only respondents (OR < 1), and for the other five items, mixed-mode respondents were more likely to select higher answer categories (OR > 1) (Additional file 4).

### Validation within the mixed-mode group

Eleven items were also answered differently by paper (n = 417) versus online (n = 369) respondents within the mixed-mode (Table 2), with two items being significant and displaying similar OR in both analyses. The calculated probability of observing the same two or more items as significantly different in both analyses is 22.4%.

**Table 2:** Results of ordinal regression analysis with a) survey design and b) survey mode as predictor

No.	Item	Adjusted^a^ OR (95% CI) mixed-mode compared to online-only	*p* value^b^ (local significance level of FDR)	Adjusted^a^ OR (95% CI) paper mode compared to online mode (within mixed-mode)	*p* value^b^ (local significance level of FDR)
12^c^	K: Relaxation exercises protect against ARI	2.12 (1.71–2.63)	10^−11^ (0.0004)	2.65 (1.96–3.58)	10^−10^ (0.0004)
25^c^	K: Avoidance of being cold protects against ARI	1.96 (1.58–2.44)	10^−9^ (0.001)	1.55 (1.16–2.07)	0.003 (0.004)
1^d^	FREQ: 12-month prevalence of infection of the upper respiratory tract	1.54 (1.26–1.88)	10^−5^ (0.001)	1.46 (1.11–1.9)	0.01 (0.005)
14^d^	K: Vitamin C protects against ARI	0.57 (0.46–0.7)	10^−7^ (0.01)	1.07 (0.82–1.41)	0.61 (0.039)
24^d^	K: Probiotic yogurt protects against ARI	1.41 (1.14–1.73)	0.001 (0.003)	1.13 (0.85–1.49)	0.41 (0.029)
26^d^	K: Cold and hot contrast showers protect against ARI	1.36 (1.11–1.67)	0.003 (0.004)	1.31 (0.99–1.72)	0.06 (0.009)
30^d^	P: Implementation of regular ventilation of living rooms	0.65 (0.52–0.82)	10^−4^ (0.002)	0.88 (0.65–1.18)	0.39 (0.028)
68^d^	A: Vaccinations are effective in preventing infectious diseases	0.66 (0.52–0.83)	0.001 (0.003)	0.65 (0.48–0.88)	0.01 (0.005)
69^d^	A: Vaccinations are getting safer and more effective	0.62 (0.5–0.78)	10^−5^ (0.002)	0.78 (0.58–1.06)	0.12 (0.015)
89^d^	K: Avoidance of woods protects against tick bites	1.37 (1.12–1.69)	0.003 (0.004)	1.21 (0.91–1.6)	0.18 (0.02)
108^d^	P: Implementation of anti-tick treatment	0.71 (0.57–0.88)	0.002 (0.003)	0.76 (0.57–1.02)	0.07 (0.01)
7^e^	FREQ: 12-month prevalence of diarrhoea	1.03 (0.84–1.27)	0.77 (0.045)	1.61 (1.23–2.13)	10^−4^ (0.002)
32^e^	P: Implementation of using saunas	1.04 (0.83–1.3)	0.71 (0.043)	0.46 (0.34–0.62)	10^−7^ (0.001)
36^e^	P: Implementation of taking homeopathic substances	0.73 (0.59–0.92)	0.01 (0.006)	0.5 (0.36–0.69)	10^−5^ (0.001)
38^e^	P: Implementation of drinking much water	0.89 (0.71–1.1)	0.28 (0.03)	0.6 (0.44–0.82)	0.001 (0.003)
40^e^	P: Implementation of outside activities	0.84 (0.68–1.04)	0.10 (0.018)	0.62 (0.46–0.82)	0.001 (0.002)
63^e^	K: Vaccination recommendation influenza	0.72 (0.54–0.96)	0.02 (0.01)	0.55 (0.36–0.82)	0.003 (0.004)
88^e^	A: Worry to get infected with Tick-borne encephalitis	1.11 (0.9–1.37)	0.32 (0.032)	1.61 (1.21–2.15)	0.001 (0.003)
103^e^	P: Implementation of avoidance of woods	1.22 (0.96–1.56)	0.11 (0.019)	1.87 (1.35–2.61)	10^−4^ (0.001)
107^e^	P: Implementation of wearing trousers in socks	0.78 (0.63–0.96)	0.02 (0.009)	0.63 (0.47–0.85)	0.002 (0.003)

Each item analysed as outcome in one ordinal regression analysis

Number of item: compare Additional file 1. *A* Question about attitudes, *ARI* acute respiratory infection, *FDR* false discovery rate, *FREQ* Question about frequency of infections, *K* Question about knowledge, *P* Question about practice

^a^Adjusted for age at baseline (fitted as fractional polynomial), sex, and highest completed education

^b^Wald test with the null hypothesis that the respective OR is equal to one

^c^OR is significantly different from one in both the comparison mixed-mode versus online-only and the comparison paper mode versus online mode

^d^OR is significantly different from one in the comparison mixed-mode versus online-only

^e^OR is significantly different from one in the comparison paper mode versus online mode

## Discussion

Using data from a population-based longitudinal panel, we investigated if an online-only study results in different response patterns when compared with a mixed-mode study. We found similar response patterns in both survey design groups with only few items being answered differently, likely due to chance. There was also no evidence of selection of respondents with respect to sociodemographic characteristics. In contrast, there were substantial differences among those choosing different modes of participation within the mixed-mode study group.

The overall response rate was slightly higher in the mixed-mode than in the online-only study. This is in line with findings of other studies [5, 6, 11]. However, the difference in response rates was smaller than the average of 11 percent points reported in a meta-analysis published in 2008 [34], which likely reflects the increasing Internet literacy over time. In the mixed-mode group, the age-specific response rate differences increased with age. In the online-only group, there were similar response rates over all age groups. While this finding might appear counterintuitive, the explanation might be that higher willingness to participate in scientific surveys compensates lower Internet literacy in older age groups. In the years to come, the expected rising Internet literacy of older age groups might increase their participation even further [35, 36]. In view of our findings, offering a choice between modes in order to slightly improve response rates does not seem to outweigh the additional effort of designing paper questionnaires, digitalising the answers, and spending money on paper and postage in the studied setting.

We found that the type of response (i.e. missing, “do not know” or valid response) did not differ significantly between the mixed-mode and the online-only group. Also the content of response (when excluding non-valid, i.e. missing and “do not know” responses) was similar between the mixed-mode and the online-only group.

Few items, i.e. 8.2% (11/134) showed significant differences in content of response between groups. Although we adjusted for sociodemographic differences (age, sex, and education), residual confounding may be an issue because we did not have information on further socioeconomic measures such as profession and equivalised disposable income. However, it is unlikely to have affected the response patterns between survey designs.

We found a difference with respect to the frequency of infections. Plante et al. [9] also found differences in response to items related to highly prevalent and nonspecific symptoms in a study comparing telephone and online data collection. All other differences observed in our study were related to prevention measures and to attitudes towards adult vaccinations. However, all other items related to preventive measures (27 items) or attitudes towards adult vaccinations (32 items) showed no significant differences among survey design groups. Therefore, it seems unlikely that an online-only study would generally attract participants with attitudes towards prevention different from those participating in a mixed-mode study.

If differences between mixed-mode and online-only were due to the online representation of the questionnaires, then these differences should also be present between the two modes of participation within the mixed-mode group and they should even be of larger magnitude. Two of the items, for which differences were observed between mixed-mode and online-only design, remained significant in the comparison within the mixed-mode group and the corresponding OR were similar in both analyses. The finding of the same two items in both analyses by chance had the probability of 22%, i.e. consistent with differences between study designs being random.

### Strengths and limitations

The strength of our study is the population-based sampling, which provides the possibility of generating generalizable estimates via post-stratification with demographical variables like age, sex, and education [37]. By inviting eligible individuals via regular mail, we likely reduced selection bias compared to convenience sampling via the Internet. Using data from our longitudinal survey, we were able to investigate response patterns related to many different topics. By applying the FDR algorithm, we controlled the chance of false positive findings, which was not done in previous studies and possibly resulted in more reported differences. We did not use the Bonferroni correction for this purpose because it is overly conservative and might not allow detecting present differences. We were able to validate our findings for response patterns both between survey designs (mixed-mode vs. online-only) and between modes of data collection within the mixed-mode. Previous studies focussed only on one of these comparisons.

Our study has also some limitations. The overall response rate was below 10%. However, response rates in epidemiologic studies in Germany are decreasing in general [38] and there is evidence that non-response bias may have little influence on estimations of relative difference between groups [39, 40].

The investigation of differences in responses in the online-only study compared to the mixed-mode group was not the primary research question of HaBIDS. As a consequence, we did not randomise participants to either get a choice between survey modes or not. We applied proportional stratified random sampling from the population registries to obtain study samples representative of the respective regions. While the regions in which different survey designs were implemented were geographically close and we did not consider randomisation necessary, we did not expect a difference in age distribution across the study regions. Since participants in the two survey design groups came from different districts, we cannot adjust for the effect of different locations, but the comparison within mixed-mode design in a single location did not indicate regional differences. Because we had to cut expenses for postage, paper-and-pencil questionnaires were sent at two time points while online questionnaires were sent at seven time points. This follow-up difference might have influenced our ability to disentangle the presence or absence of mode differences.

## Conclusions

Data collected in the online-only group of our study were largely comparable to data from mixed-mode group, where participants had a choice between modes. With increasing use of the Internet, online-only studies offer a cheaper alternative to mixed-mode studies. Our study confirms that such an approach is not suffering from biased estimates caused by excluding individuals who would participate only in a paper-and-pencil study.

## Additional files

**Additional file 1.** Items used in the analyses and level of measurement.

**Additional file 2.** Age distributions of the two samples obtained from the population registries and for the entire population of Lower Saxony by 9 May 2011.

**Additional file 3.** Results of multinomial logistic regression analysis of type of response with study design group as independent variable (reference: online-only).

**Additional file 4.** Results of ordinal regression analysis with survey design group as predictor (reference: online-only) for response to an item (each item analysed as outcome in one ordinal regression analysis).

## Abbreviations

CI: confidence interval
FDR: false discovery rate
KAP: knowledge-attitude-practice
OR: odds ratio
