# Supplementary material for: Comparison of response patterns in different survey designs: a longitudinal panel with mixed-mode and online-only design
Source: Emerg Themes Epidemiol. 2017 Mar 21;14:4. doi: 10.1186/s12982-017-0058-2 (PMC5361716; doi:10.1186/s12982-017-0058-2)
Supplement: Supplementary file 1 — Additional file 1. Items used in the analyses and level of measurement. [file 12982_2017_58_MOESM1_ESM.docx]

Additional file 1 - Items used in the analyses and level of measurement

| **No.** | **Item** | **Level of measurement** |
| --- | --- | --- |
| Frequency of infections and infection-associated symptoms in the last 12 months | | |
| 1 | FREQ: 12-month prevalence of infection of the upper respiratory tract | ordinal  (from 0=”never” to  5=”more than 6 times”;  90=”do not know”) |
| 2 | FREQ: 12-month prevalence of infection of the lower respiratory tract |  |
| 3 | FREQ: 12-month prevalence of bladder infection |  |
| 4 | FREQ: 12-month prevalence of orolabial herpes |  |
| 5 | FREQ: 12-month prevalence of cough lasting more than 4 weeks |  |
| 6 | FREQ: 12-month prevalence of fever |  |
| 7 | FREQ: 12-month prevalence of diarrhoea |  |
| 8 | FREQ: Life-time prevalence of herpes zoster |  |
| Health and psychosocial factors | | |
| 9 | X: Self-rated health | ordinal  (from 0=”bad” to  5=”excellent”) |
| 10 | X: Unusual exhaustion and fatigue in the past 7 days | ordinal  (from 0=”not at all” to  10=”very strong”) |
| Prevention measures against respiratory infections | | |
| 11 | K: Thorough hand washing with soap protects against ARI | ordinal  (from 0=”does not protect at all” to  4=”protects very well”;  90=”do not know”) |
| 12 | K: Relaxation exercises protect against ARI |  |
| 13 | K: Regular ventilation of living rooms protects against ARI |  |
| 14 | K: Vitamin C protects against ARI |  |
| 15 | K: Using saunas protects against ARI |  |
| 16 | K: Engaging in endurance sports protects against ARI |  |
| 17 | K: Nasal douches protect against ARI |  |
| 18 | K: Healthy diet, eating many fruits and vegetables protect against ARI |  |
| 19 | K: Homeopathic substances protect against ARI |  |
| 20 | K: Avoidance of overheating of living rooms protects against ARI |  |
| 21 | K: Drinking much water protects against ARI |  |
| 22 | K: Enough sleep protects against ARI |  |
| 23 | K: Outside activities protect against ARI |  |
| 24 | K: Probiotic yogurt protects against ARI |  |
| 25 | K: Avoidance of being cold protects against ARI |  |
| 26 | K: Cold and hot contrast showers protect against ARI |  |
| 27 | K: Avoidance of contact to sick people protects against ARI |  |
| 28 | P: Implementation of thorough hand washing with soap | ordinal  (from 0=”never” to  5=”several times a day”) |
| 29 | P: Implementation of relaxation exercises |  |
| 30 | P: Implementation of regular ventilation of living rooms |  |
| 31 | P: Implementation of taking Vitamin C |  |
| 32 | P: Implementation of using saunas |  |
| 33 | P: Implementation of engaging in endurance sports |  |
| 34 | P: Implementation of nasal douches |  |
| 35 | P: Implementation of healthy diet, eating many fruits and vegetables |  |
| 36 | P: Implementation of taking homeopathic substances |  |
| 37 | P: Implementation of avoidance of overheating of living rooms |  |
| 38 | P: Implementation of drinking much water |  |
| 39 | P: Implementation of sleeping enough |  |
| 40 | P: Implementation of outside activities |  |
| 41 | P: Implementation of eating probiotic yogurt |  |
| 42 | P: Implementation of avoidance of being cold |  |
| 43 | P: Implementation of cold and hot contrast showers |  |
| 44 | P: Implementation of avoidance of contact to sick people |  |
| 45 | P: Different preventive behaviour during winter season | binary  (0=”no”; 1=”yes”) |
| Vaccinations | | |
| 46 | P: Vaccination against diphtheria in the past 10 years | binary  (0=”no”; 1=”yes”;  90=”do not know”) |
| 47 | P: Vaccination against tetanus in the past 10 years |  |
| 48 | P: Vaccination against pertussis in the past 10 years |  |
| 49 | P: Vaccination against pneumococcus in the past 10 years |  |
| 50 | P: Vaccination against poliomyelitis in the past 10 years |  |
| 51 | P: Vaccination against hepatitis B in the past 10 years |  |
| 52 | A: Intended vaccination against diphtheria (in the future) |  |
| 53 | A: Intended vaccination against tetanus (in the future) |  |
| 54 | A: Intended vaccination against pertussis (in the future) |  |
| 55 | A: Intended vaccination against pneumococcus (in the future) |  |
| 56 | A: Intended vaccination against poliomyelitis (in the future) |  |
| 57 | A: Intended vaccination against hepatitis B (in the future) |  |
| 58 | P: Frequency of influenza vaccinations in the past 10 years | ordinal  (from 0=”never” to  5=”every year”;  90=”do not know”) |
| 59 | A: Intended vaccination against influenza (in the future) | binary  (0=”no”; 1=”yes”;  90=”do not know”) |
| 60 | P: Vaccination against influenza H1N1 in the winter season 2009/2010 |  |
| 61 | K: Vaccination recommendation diphtheria | binary  (0=”is not true”; 1=”is true”;  90=”do not know”) |
| 62 | K: Vaccination recommendation pertussis |  |
| 63 | K: Vaccination recommendation measles |  |
| 64 | K: Vaccination recommendation influenza |  |
| 65 | K: Vaccination recommendation pneumococcus |  |
| 66 | K: Vaccination recommendation poliomyelitis |  |
| 67 | K: Vaccination recommendation rabies |  |
| 68 | A: Vaccinations are effective in preventing infectious diseases | ordinal  (from 0=”I do not agree” to  3=”I totally agree”;  90=”do not know”) |
| 69 | A: Vaccinations are getting safer and more effective |  |
| 70 | A: Immune system is weakened because of to many vaccinations |  |
| 71 | A: No vaccination because being scared of the syringes |  |
| 72 | A: No vaccination because being scared of the adverse effects |  |
| 73 | A: No vaccination because being scared of the late effects |  |
| 74 | A: Compulsory vaccination for all adults |  |
| 75 | A: Compulsory vaccination for medical staff |  |
| 76 | A: Vaccinations in general | ordinal  (from 0=”negative” to  3=”positive”;  90=”do not know”) |
| 77 | A: Vaccination against tetanus |  |
| 78 | A: Vaccination against influenza |  |
| 79 | K: Ever heard of human papillomavirus | binary  (0=”no”; 1=”yes”;  90=”do not know”) |
| Tick-borne infections | | |
| 80 | P: Frequency of private stays in the woods | ordinal  (from 0=”never” to  5=”several times a day”) |
| 81 | K: Ticks transmit Borreliosis | ordinal  (from 0=”is not true” to  3=”is true”;  90=”do not know”) |
| 82 | K: Borreliosis is a serious disease |  |
| 83 | K: Children are particularly vulnerable to borreliosis |  |
| 84 | A: Worry to get infected with borreliosis |  |
| 85 | K: Ticks transmit tick-borne encephalitis (TBE) |  |
| 86 | K: TBE is a serious disease |  |
| 87 | K: Children are particularly vulnerable to TBE |  |
| 88 | A: Worry to get infected with TBE |  |
| 89 | K: Avoidance of woods protects against tick bites | ordinal  (from 0=”does not protect at all” to  3=”protects very well”;  90=”do not know”) |
| 90 | K: Avoidance of meadows protects against tick bites |  |
| 91 | K: Long clothes protects against tick bites |  |
| 92 | K: Ankle-high shoes protects against tick bites |  |
| 93 | K: Wearing trousers in socks protects against tick bites |  |
| 94 | K: Anti-tick treatment protects against tick bites |  |
| 95 | K: Inspection of the body after stays in the woods protects against tick bites |  |
| 96 | K: After a tick bite: remove the tick immediately | ordinal  (from 0=”not important” to  3=”very important”;  90=”do not know”) |
| 97 | K: After a tick bite: a doctor should remove the tick |  |
| 98 | K: After a tick bite: apply alcoholic solution on the tick before removing it |  |
| 99 | K: After a tick bite: apply toothpaste on the tick before removing it |  |
| 100 | K: After a tick bite: apply oil on the tick before removing it |  |
| 101 | K: After a tick bite: remove the tick by pulling it straight |  |
| 102 | K: After a tick bite: unscrew the tick |  |
| 103 | P: Implementation of avoidance of woods | ordinal  (from 0=”never” to  3=”always”) |
| 104 | P: Implementation of avoidance of meadows |  |
| 105 | P: Implementation of long clothes |  |
| 106 | P: Implementation of ankle-high shoes |  |
| 107 | P: Implementation of wearing trousers in socks |  |
| 108 | P: Implementation of anti-tick treatment |  |
| 109 | P: Implementation of inspection of the body after stays in the woods |  |
| 110 | P: Implementation of removing the tick immediately | ordinal  (from 0=”in no case”  to 3=”in any case”) |
| 111 | P: Implementation of removing the tick at a doctor's office |  |
| 112 | P: Implementation of applying alcoholic solution on the tick before removing it |  |
| 113 | P: Implementation of applying toothpaste on the tick before removing it |  |
| 114 | P: Implementation of applying oil on the tick before removing it |  |
| 115 | P: Implementation of removing the tick by pulling it straight |  |
| 116 | P: Implementation of unscrewing the tick |  |
| Antibiotics | | |
| 117 | K: Antibiotics are effective against bacteria | ordinal  (from 0=”is not true” to  3=”is true”) |
| 118 | K: Antibiotics are effective against viruses |  |
| 119 | K: Penicillin is an antibiotic |  |
| 120 | K: Paracetamol is an antibiotic |  |
| 121 | K: Ibuprofen is an antibiotic |  |
| 122 | K: If an antibiotic is not taken as prescribed, then germs become resistant |  |
| 123 | K: If someone takes often antibiotics, then her/his body becomes immune |  |
| 124 | P: Asked GP for antibiotics because of a cold |  |
| 125 | P: Antibiotics in stock at home |  |
| 126 | P: Taking antibiotics according to recommendation |  |
| 127 | P: Stop antibiotic therapy when feeling better |  |
| 128 | P: Share the antibiotics with relatives |  |
| 129 | P: No intake of antibiotics at all |  |
| 130 | A: Worry about antibiotic resistances |  |
| 131 | P: Last time taking antibiotics:  asked for the antibiotic |  |
| 132 | P: Last time taking antibiotics:  according to recommended number of pills |  |
| 133 | P: Last time taking antibiotics:  according to recommended time interval of intake |  |
| 134 | P: Last time taking antibiotics:  according to recommended duration of antibiotic intake |  |

A: Question about attitudes

ARI: Acute respiratory infection

FREQ: Question about frequency of infections

GP: General practitioner

K: Question about knowledge

P: Question about practice

X: Question about well-being
