# Supplementary material for: Comparison of response patterns in different survey designs: a longitudinal panel with mixed-mode and online-only design
Source: Emerg Themes Epidemiol. 2017 Mar 21;14:4. doi: 10.1186/s12982-017-0058-2 (PMC5361716; doi:10.1186/s12982-017-0058-2)
Supplement: Supplementary file 2 — Additional file 2. Age distributions of the two samples obtained from the population registries and for the entire population of Lower Saxony by 9 May 2011. [file 12982_2017_58_MOESM2_ESM.docx]

Additional file 2 - Age distributions of the two samples obtained from the population registries and for the entire population of Lower Saxony by 9 May 2011

| **Age** | **Invited to**  **mixed-mode survey,**  **n (%)** | **Invited to**  **online-only survey,**  **n (%)** | **Population of**  **Lower Saxony,**  **n (%)** |
| --- | --- | --- | --- |
| 15 – 19 years | 1179  (7.4%) | 775  (7.7%) | 436388  (8.0%) |
| 20 – 24 years | 1534  (9.6%) | 857  (8.6%) | 446460  (8.2%) |
| 25 – 29 years | 1598  (10.0%) | 774  (7.7%) | 422659  (7.7%) |
| 30 – 34 years | 1516  (9.5%) | 748  (7.5%) | 422530  (7.7%) |
| 35 – 39 years | 1392  (8.7%) | 737  (7.4%) | 452754  (8.3%) |
| 40 – 44 years | 1674  (10.5%) | 1006  (10.1%) | 632918  (11.6%) |
| 45 – 49 years | 1871  (11.7%) | 1222  (12.2%) | 680386  (12.4%) |
| 50 – 54 years | 1690  (10.6%) | 1134  (11.3%) | 593993  (10.9%) |
| 55 – 59 years | 1394  (8.7%) | 1005  (10.0%) | 510476  (9.3%) |
| 60 – 64 years | 1209  (7.6%) | 943  (9.4%) | 464803  (8.5%) |
| 65 – 69 years | 944  (5.9%) | 801  (8.0%) | 404087  (7.4%) |
