# Supplementary material for: Comparison of response patterns in different survey designs: a longitudinal panel with mixed-mode and online-only design
Source: Emerg Themes Epidemiol. 2017 Mar 21;14:4. doi: 10.1186/s12982-017-0058-2 (PMC5361716; doi:10.1186/s12982-017-0058-2)
Supplement: Supplementary file 3 — Additional file 3. Results of multinomial logistic regression analysis of type of response with study design group as independent variable (reference: online-only). [file 12982_2017_58_MOESM3_ESM.docx]

Additional file 3 - Results of multinomial logistic regression analysis of type of response with study design group as independent variable (reference: online-only)

| **No.** | **Item** | **P value^a^** | **P value^b^** | **Local signifi-cance level of FDR** |
| --- | --- | --- | --- | --- |
| Frequency of infections and infection-associated symptoms in the last 12 months | | | | |
| 1 | FREQ: 12-month prevalence of infection of the upper respiratory tract | <0.001 | 0.33 | 0.03 |
| 2 | FREQ: 12-month prevalence of infection of the lower respiratory tract | <0.001 | 0.03 | 0.007 |
| 3 | FREQ: 12-month prevalence of bladder infection | <0.001 | 0.02 | 0.006 |
| 4 | FREQ: 12-month prevalence of orolabial herpes | <0.001 | 0.65 | 0.04 |
| 5 | FREQ: 12-month prevalence of cough lasting more than 4 weeks | <0.001 | 0.33 | 0.03 |
| 6 | FREQ: 12-month prevalence of fever | <0.001 | 0.20 | 0.03 |
| 7 | FREQ: 12-month prevalence of diarrhoea | <0.001 | 0.53 | 0.04 |
| 8 | FREQ: Life-time prevalence of herpes zoster | <0.001 | 0.03 | 0.009 |
| Health and sociodemographic factors | | | | |
| 9 | X: Self-rated health | <0.001 | 0.08 | 0.02 |
| 10 | X: Unusual exhaustion and fatigue in the past 7 days | <0.001 | 0.66 | 0.04 |
| Prevention measures against respiratory infections | | | | |
| 11 | K: Thorough hand washing with soap protects against ARI | 0.45 | 0.59 | 0.04 |
| 12 | K: Relaxation exercises protect against ARI | 0.82 | 0.95 | 0.05 |
| 13 | K: Regular ventilation of living rooms protects against ARI | 0.07 | 0.06 | 0.01 |
| 14 | K: Vitamin C protects against ARI | 0.63 | 0.22 | 0.03 |
| 15 | K: Using saunas protects against ARI | 0.25 | 0.12 | 0.02 |
| 16 | K: Engaging in endurance sports protects against ARI | 0.81 | 0.82 | 0.05 |
| 17 | K: Nasal douches protect against ARI | 0.02 | <0.001 | 0.003 |
| 18 | K: Healthy diet and eating many fruits and vegetables protect against ARI | 0.07 | 0.11 | 0.02 |
| 19 | **K: Homeopathic substances protect against ARI** | <0.001 | **<0.001** | 0.0004 |
| 20 | K: Avoidance of overheating of living rooms protects against ARI | 0.21 | 0.28 | 0.03 |
| 21 | K: Drinking much water protects against ARI | 0.45 | 0.13 | 0.02 |
| 22 | K: Enough sleep protects against ARI | 0.82 | 0.56 | 0.04 |
| 23 | K: Outside activities protect against ARI | 0.54 | 0.48 | 0.04 |
| 24 | K: Probiotic yogurt protects against ARI | 0.58 | 0.65 | 0.04 |
| 25 | K: Avoidance of being cold protects against ARI | 0.91 | 0.70 | 0.04 |
| 26 | K: Cold and hot contrast showers protect against ARI | 0.48 | 0.70 | 0.04 |
| 27 | K: Avoidance of contact to sick people protects against ARI | 0.84 | 0.35 | 0.03 |
| 28 | P: Implementation of thorough hand washing with soap | 0.04 | 0.05 | 0.01 |
| 29 | P: Implementation of relaxation exercises | 0.05 | 0.05 | 0.01 |
| 30 | P: Implementation of regular ventilation of living rooms | 0.02 | 0.02 | 0.005 |
| 31 | P: Implementation of taking Vitamin C | 0.64 | 0.75 | 0.04 |
| 32 | P: Implementation of using saunas | 0.09 | 0.12 | 0.02 |
| 33 | P: Implementation of engaging in endurance sports | 0.29 | 0.36 | 0.03 |
| 34 | P: Implementation of nasal douches | 0.29 | 0.38 | 0.03 |
| 35 | P: Implementation of healthy diet and eating many fruits and vegetables | 0.09 | 0.09 | 0.02 |
| 36 | P: Implementation of taking homeopathic substances | 0.43 | 0.50 | 0.04 |
| 37 | P: Implementation of avoidance of overheating of living rooms | 0.44 | 0.43 | 0.03 |
| 38 | P: Implementation of drinking much water | 0.09 | 0.09 | 0.02 |
| 39 | P: Implementation of sleeping enough | 0.12 | 0.13 | 0.02 |
| 40 | P: Implementation of outside activities | 0.02 | 0.03 | 0.009 |
| 41 | P: Implementation of eating probiotic yogurt | 0.02 | 0.03 | 0.007 |
| 42 | P: Implementation of avoidance of being cold | 0.12 | 0.15 | 0.02 |
| 43 | P: Implementation of cold and hot contrast showers | 0.82 | 0.87 | 0.05 |
| 44 | P: Implementation of avoidance of contact to sick people | 0.02 | 0.03 | 0.008 |
| 45 | P: Different preventive behaviour during winter season | 0.04 | 0.06 | 0.01 |
| Vaccinations | | | | |
| 46 | P: Vaccination against diphtheria in the past 10 years | 0.02 | 0.01 | 0.005 |
| 47 | P: Vaccination against tetanus in the past 10 years | 0.03 | 0.08 | 0.02 |
| 48 | P: Vaccination against pertussis in the past 10 years | 0.03 | 0.01 | 0.004 |
| 49 | P: Vaccination against pneumococcus in the past 10 years | 0.04 | 0.04 | 0.009 |
| 50 | P: Vaccination against poliomyelitis in the past 10 years | 0.20 | 0.10 | 0.02 |
| 51 | P: Vaccination against hepatitis B in the past 10 years | 0.06 | 0.02 | 0.006 |
| 52 | A: Intended vaccination against diphtheria (in the future) | 0.12 | 0.08 | 0.02 |
| 53 | A: Intended vaccination against tetanus (in the future) | 0.01 | 0.02 | 0.007 |
| 54 | A: Intended vaccination against pertussis (in the future) | 0.14 | 0.04 | 0.01 |
| 55 | A: Intended vaccination against pneumococcus (in the future) | 0.16 | 0.06 | 0.01 |
| 56 | A: Intended vaccination against poliomyelitis (in the future) | 0.11 | 0.05 | 0.01 |
| 57 | A: Intended vaccination against hepatitis B (in the future) | 0.35 | 0.21 | 0.03 |
| 58 | P: Frequency of influenza vaccinations in the past 10 years | 0.34 | 0.21 | 0.03 |
| 59 | A: Intended vaccination against influenza (in the future) | 0.96 | 0.99 | 0.05 |
| 60 | P: Vaccination against influenza H1N1 in the winter season 2009/2010 | 0.40 | 0.47 | 0.03 |
| 61 | K: Vaccination recommendation diphtheria | 0.58 | 0.42 | 0.04 |
| 62 | K: Vaccination recommendation pertussis | 0.98 | 0.96 | 0.05 |
| 63 | K: Vaccination recommendation measles | 0.62 | 0.55 | 0.04 |
| 64 | K: Vaccination recommendation influenza | 0.44 | 0.60 | 0.04 |
| 65 | K: Vaccination recommendation pneumococcus | 0.56 | 0.58 | 0.04 |
| 66 | K: Vaccination recommendation poliomyelitis | 0.47 | 0.54 | 0.04 |
| 67 | K: Vaccination recommendation rabies | 0.91 | 0.94 | 0.05 |
| 68 | A: Vaccinations are effective in preventing infectious diseases | 0.33 | 0.32 | 0.03 |
| 69 | A: Vaccinations are getting safer and more effective | 0.38 | 0.41 | 0.03 |
| 70 | A: Immune system is weakened because of to many vaccinations | 0.40 | 0.46 | 0.03 |
| 71 | A: No vaccination because being scared of the syringes | 0.40 | 0.55 | 0.04 |
| 72 | A: No vaccination because being scared of the adverse effects | 0.30 | 0.35 | 0.03 |
| 73 | A: No vaccination because being scared of the late effects | 0.70 | 0.71 | 0.04 |
| 74 | A: Compulsory vaccination for all adults | 0.31 | 0.33 | 0.03 |
| 75 | A: Compulsory vaccination for medical staff | 0.14 | 0.19 | 0.03 |
| 76 | A: Vaccinations in general | 0.74 | 0.78 | 0.05 |
| 77 | A: Vaccination against tetanus | 0.90 | 0.94 | 0.05 |
| 78 | A: Vaccination against influenza | 0.19 | 0.11 | 0.02 |
| 79 | K: Ever heard of human papillomavirus | 0.18 | 0.42 | 0.03 |
| Tick-borne infections | | | | |
| 80 | P: Frequency of private stays in the woods | 0.41 | 0.43 | 0.03 |
| 81 | K: Ticks transmit Borreliosis | 0.54 | 0.83 | 0.05 |
| 82 | K: Borreliosis is a serious disease | 0.84 | 0.76 | 0.04 |
| 83 | K: Children are particularly vulnerable to borreliosis | 0.57 | 0.50 | 0.04 |
| 84 | A: Worry to get infected with borreliosis | 0.31 | 0.24 | 0.03 |
| 85 | K: Ticks transmit tick-borne encephalitis (TBE) | 0.66 | 0.87 | 0.05 |
| 86 | K: TBE is a serious disease | 0.70 | 0.89 | 0.05 |
| 87 | K: Children are particularly vulnerable to TBE | 0.39 | 0.20 | 0.03 |
| 88 | A: Worry to get infected with TBE | 0.10 | 0.11 | 0.02 |
| 89 | K: Avoidance of woods protects against tick bites | 0.10 | 0.02 | 0.006 |
| 90 | K: Avoidance of meadows protects against tick bites | 0.24 | 0.17 | 0.02 |
| 91 | K: Long clothes protects against tick bites | 0.14 | 0.16 | 0.02 |
| 92 | K: Ankle-high shoes protects against tick bites | 0.43 | 0.51 | 0.04 |
| 93 | K: Wearing trousers in socks protects against tick bites | 0.10 | 0.05 | 0.01 |
| 94 | K: Anti-tick treatment protects against tick bites | 0.12 | 0.08 | 0.01 |
| 95 | K: Inspection of the body after stays in the woods protects against tick bites | 0.05 | 0.08 | 0.01 |
| 96 | K: After a tick bite: remove the tick immediately | 0.38 | 0.55 | 0.04 |
| 97 | K: After a tick bite: a doctor should remove the tick | 0.01 | 0.01 | 0.004 |
| 98 | K: After a tick bite: apply alcoholic solution on the tick before removing it | 0.06 | 0.08 | 0.02 |
| 99 | K: After a tick bite: apply toothpaste on the tick before removing it | 0.01 | 0.01 | 0.004 |
| 100 | K: After a tick bite: apply oil on the tick before removing it | 0.07 | 0.08 | 0.01 |
| 101 | K: After a tick bite: remove the tick by pulling it straight | <0.001 | <0.001 | 0.002 |
| 102 | K: After a tick bite: unscrew the tick | <0.001 | <0.001 | 0.002 |
| 103 | P: Implementation of avoidance of woods | <0.001 | <0.001 | 0.001 |
| 104 | P: Implementation of avoidance of meadows | 0.01 | 0.01 | 0.003 |
| 105 | P: Implementation of long clothes | 0.56 | 0.62 | 0.04 |
| 106 | P: Implementation of ankle-high shoes | 0.23 | 0.22 | 0.03 |
| 107 | P: Implementation of wearing trousers in socks | 0.06 | 0.06 | 0.01 |
| 108 | P: Implementation of anti-tick treatment | 0.20 | 0.08 | 0.02 |
| 109 | P: Implementation of inspection of the body after stays in the woods | 0.03 | 0.03 | 0.008 |
| 110 | P: Implementation of removing the tick immediately | 0.18 | 0.13 | 0.02 |
| 111 | P: Implementation of removing the tick at a doctor's office | <0.001 | <0.001 | 0.001 |
| 112 | P: Implementation of applying alcoholic solution on the tick before removing it | 0.07 | 0.07 | 0.01 |
| 113 | P: Implementation of applying toothpaste on the tick before removing it | 0.11 | 0.11 | 0.02 |
| 114 | P: Implementation of applying oil on the tick before removing it | 0.20 | 0.18 | 0.02 |
| 115 | P: Implementation of removing the tick by pulling it straight | <0.001 | <0.001 | 0.001 |
| 116 | P: Implementation of unscrewing the tick | 0.01 | <0.001 | 0.003 |
| Antibiotics | | | | |
| 117 | K: Antibiotics are effective against bacteria | 0.46 | 0.70 | 0.04 |
| 118 | K: Antibiotics are effective against viruses | 0.36 | 0.47 | 0.03 |
| 119 | K: Penicillin is an antibiotic | 0.87 | 0.86 | 0.05 |
| 120 | K: Paracetamol is an antibiotic | 0.37 | 0.30 | 0.03 |
| 121 | K: Ibuprofen is an antibiotic | 0.66 | 0.45 | 0.03 |
| 122 | K: If an antibiotic is not taken as prescribed, then germs become resistant | 0.07 | 0.07 | 0.01 |
| 123 | K: If someone takes often antibiotics, then her/his body becomes immune | 0.17 | 0.16 | 0.02 |
| 124 | P: Asked GP for antibiotics because of a cold | 0.22 | 0.20 | 0.03 |
| 125 | P: Antibiotics in stock at home | 0.15 | 0.12 | 0.02 |
| 126 | P: Taking antibiotics according to recommendation | 0.74 | 0.61 | 0.04 |
| 127 | P: Stop antibiotic therapy when feeling better | 0.87 | 0.77 | 0.04 |
| 128 | P: Share the antibiotics with relatives | 0.79 | 0.90 | 0.05 |
| 129 | P: No intake of antibiotics at all | 0.89 | 0.86 | 0.05 |
| 130 | A: Worry about antibiotic resistances | 0.60 | 0.69 | 0.04 |
| 131 | P: Last time taking antibiotics:  asked for the antibiotic | 0.18 | 0.13 | 0.02 |
| 132 | P: Last time taking antibiotics:  according to recommended number of pills | 0.11 | 0.10 | 0.02 |
| 133 | P: Last time taking antibiotics:  according to recommended time interval of intake | 0.10 | 0.08 | 0.02 |
| 134 | P: Last time taking antibiotics:  according to recommended duration of antibiotic intake | 0.08 | 0.06 | 0.01 |

^a^Likelihood ratio test of the respective model with survey design group included as independent variable versus empty model

^b^Likelihood ratio test of the respective model with survey design group, age, sex, and education included as independent variables versus the model with only age, sex, and education as independent variables

Bold: items with p value^b^ significant after controlling the FDR

A: Question about attitudes

ARI: Acute respiratory infection

FDR: False discovery rate

FREQ: Question about frequency of infections

GP: General practitioner

K: Question about knowledge

P: Question about practice

X: Question about well-being
