# Supplementary material for: Comparison of response patterns in different survey designs: a longitudinal panel with mixed-mode and online-only design
Source: Emerg Themes Epidemiol. 2017 Mar 21;14:4. doi: 10.1186/s12982-017-0058-2 (PMC5361716; doi:10.1186/s12982-017-0058-2)
Supplement: Supplementary file 4 — Additional file 4. Results of ordinal regression analysis with survey design group as predictor (reference: online-only) for response to an item (each item analysed as outcome in one ordinal regression analysis). [file 12982_2017_58_MOESM4_ESM.docx]

Additional file 4 - Results of ordinal regression analysis with survey design group as predictor (reference: online-only) for response to an item (each item analysed as outcome in one ordinal regression analysis)

| **Nr** | **Item** | **Unadjusted OR**  **(95% CI)**  **mixed-mode compared to online-only** | **P value^a^** | **Adjusted^b^ OR**  **(95% CI)**  **mixed-mode compared to online-only** | **P value^a^** | **Local signifi-cance level of FDR** |
| --- | --- | --- | --- | --- | --- | --- |
| Frequency of infections and infection-associated symptoms in the last 12 months | | | | | | |
| 1 | **FREQ: 12-month prevalence of infection of the upper respiratory tract** | 1.62  (1.33-1.97) | <0.001 | **1.54**  **(1.26-1.88)** | **<0.001** | 0.001 |
| 2 | FREQ: 12-month prevalence of infection of the lower respiratory tract | 1.31  (1.01-1.71) | 0.04 | 1.36  (1.04-1.77) | 0.03 | 0.01 |
| 3 | FREQ: 12-month prevalence of bladder infection | 1.01  (0.72-1.43) | 0.95 | 1.04  (0.73-1.49) | 0.83 | 0.05 |
| 4 | FREQ: 12-month prevalence of orolabial herpes | 1.06  (0.84-1.34) | 0.61 | 1.08  (0.85-1.37) | 0.52 | 0.04 |
| 5 | FREQ: 12-month prevalence of cough lasting more than 4 weeks | 1.15  (0.88-1.49) | 0.31 | 1.17  (0.9-1.53) | 0.25 | 0.03 |
| 6 | FREQ: 12-month prevalence of fever | 1.09  (0.85-1.39) | 0.49 | 1.05  (0.82-1.35) | 0.68 | 0.04 |
| 7 | FREQ: 12-month prevalence of diarrhoea | 1.02  (0.83-1.25) | 0.87 | 1.03  (0.84-1.27) | 0.77 | 0.04 |
| 8 | FREQ: Life-time prevalence of herpes zoster | 0.66  (0.47-0.91) | 0.01 | 0.67  (0.48-0.94) | 0.02 | 0.009 |
| Health and sociodemographic factors | | | | | | |
| 9 | X: Self-rated health | 0.92  (0.75-1.13) | 0.41 | 0.83  (0.67-1.02) | 0.08 | 0.02 |
| 10 | X: Unusual exhaustion and fatigue in the past 7 days | 1.29  (1.07-1.56) | 0.01 | 1.25  (1.03-1.51) | 0.03 | 0.01 |
| Prevention measures against respiratory infections | | | | | | |
| 11 | K: Thorough hand washing with soap protects against ARI | 0.88  (0.71-1.08) | 0.21 | 0.91  (0.73-1.12) | 0.36 | 0.03 |
| 12 | **K: Relaxation exercises protect against ARI** | 2.06  (1.67-2.54) | <0.001 | **2.12**  **(1.71-2.63)** | **<0.001** | 0.0004 |
| 13 | K: Regular ventilation of living rooms protects against ARI | 1.08  (0.88-1.32) | 0.46 | 1.13  (0.92-1.39) | 0.23 | 0.03 |
| 14 | **K: Vitamin C protects against ARI** | 0.58  (0.48-0.72) | <0.001 | **0.57**  **(0.46-0.7)** | **<0.001** | 0.001 |
| 15 | K: Using saunas protects against ARI | 1.16  (0.94-1.43) | 0.17 | 1.18  (0.95-1.46) | 0.14 | 0.02 |
| 16 | K: Engaging in endurance sports protects against ARI | 1.16  (0.95-1.43) | 0.15 | 1.14  (0.92-1.41) | 0.23 | 0.03 |
| 17 | K: Nasal douches protect against ARI | 0.98  (0.79-1.21) | 0.83 | 0.98  (0.79-1.23) | 0.88 | 0.05 |
| 18 | K: Healthy diet, eating many fruits and vegetables protect against ARI | 1.36  (1.11-1.67) | <0.001 | 1.33  (1.08-1.64) | 0.01 | 0.006 |
| 19 | K: Homeopathic substances protect against ARI | 0.75  (0.6-0.93) | 0.01 | 0.83  (0.66-1.04) | 0.11 | 0.02 |
| 20 | K: Avoidance of overheating of living rooms protects against ARI | 1.08  (0.88-1.33) | 0.44 | 1.1  (0.89-1.36) | 0.39 | 0.04 |
| 21 | K: Drinking much water protects against ARI | 0.9  (0.72-1.12) | 0.33 | 0.96  (0.77-1.2) | 0.73 | 0.04 |
| 22 | K: Enough sleep protects against ARI | 1.23  (1-1.51) | 0.05 | 1.34  (1.08-1.66) | 0.01 | 0.006 |
| 23 | K: Outside activities protect against ARI | 1.05  (0.85-1.29) | 0.64 | 1.11  (0.9-1.38) | 0.32 | 0.03 |
| 24 | **K: Probiotic yogurt protects against ARI** | 1.4  (1.14-1.71) | <0.001 | **1.41**  **(1.14-1.73)** | **<0.001** | 0.003 |
| 25 | **K: Avoidance of being cold protects against ARI** | 2  (1.62-2.47) | <0.001 | **1.96**  **(1.58-2.44)** | **<0.001** | 0.001 |
| 26 | **K: Cold and hot contrast showers protect against ARI** | 1.37  (1.12-1.67) | <0.001 | **1.36**  **(1.11-1.67)** | **<0.001** | 0.004 |
| 27 | K: Avoidance of contact to sick people protects against ARI | 0.84  (0.68-1.03) | 0.09 | 0.82  (0.66-1.02) | 0.07 | 0.02 |
| 28 | P: Implementation of thorough hand washing with soap | 0.67  (0.41-1.1) | 0.11 | 0.74  (0.44-1.25) | 0.26 | 0.03 |
| 29 | P: Implementation of relaxation exercises | 1.09  (0.89-1.32) | 0.41 | 1.17  (0.95-1.43) | 0.13 | 0.02 |
| 30 | **P: Implementation of regular ventilation of living rooms** | 0.62  (0.5-0.77) | <0.001 | **0.65**  **(0.52-0.82)** | **<0.001** | 0.002 |
| 31 | P: Implementation of taking Vitamin C | 0.8  (0.65-0.99) | 0.04 | 0.83  (0.67-1.02) | 0.08 | 0.02 |
| 32 | P: Implementation of using saunas | 1.07  (0.86-1.32) | 0.56 | 1.04  (0.83-1.3) | 0.71 | 0.04 |
| 33 | P: Implementation of engaging in endurance sports | 0.89  (0.74-1.08) | 0.23 | 0.87  (0.72-1.06) | 0.16 | 0.02 |
| 34 | P: Implementation of nasal douches | 0.88  (0.67-1.14) | 0.33 | 0.88  (0.67-1.15) | 0.35 | 0.03 |
| 35 | P: Implementation of healthy diet, eating many fruits and vegetables | 0.89  (0.73-1.08) | 0.22 | 0.9  (0.73-1.1) | 0.29 | 0.03 |
| 36 | P: Implementation of taking homeopathic substances | 0.75  (0.61-0.94) | 0.01 | 0.73  (0.59-0.92) | 0.01 | 0.006 |
| 37 | P: Implementation of avoidance of overheating of living rooms | 1.04  (0.85-1.26) | 0.73 | 1.08  (0.88-1.31) | 0.46 | 0.04 |
| 38 | P: Implementation of drinking much water | 0.85  (0.69-1.06) | 0.15 | 0.89  (0.71-1.1) | 0.28 | 0.03 |
| 39 | P: Implementation of sleeping enough | 1.02  (0.82-1.25) | 0.88 | 1.1  (0.89-1.37) | 0.37 | 0.03 |
| 40 | P: Implementation of outside activities | 0.85  (0.69-1.04) | 0.12 | 0.84  (0.68-1.04) | 0.10 | 0.02 |
| 41 | P: Implementation of eating probiotic yogurt | 0.94  (0.78-1.15) | 0.57 | 0.93  (0.76-1.14) | 0.48 | 0.04 |
| 42 | P: Implementation of avoidance of being cold | 0.83  (0.68-1) | 0.05 | 0.88  (0.72-1.07) | 0.20 | 0.03 |
| 43 | P: Implementation of cold and hot contrast showers | 1.19  (0.99-1.45) | 0.07 | 1.25  (1.02-1.52) | 0.03 | 0.01 |
| 44 | P: Implementation of avoidance of contact to sick people | 0.85  (0.7-1.03) | 0.10 | 0.93  (0.76-1.13) | 0.44 | 0.04 |
| 45 | P: Different preventive behaviour during winter season | 0.75  (0.6-0.94) | 0.01 | 0.75  (0.6-0.93) | 0.01 | 0.007 |
| Vaccinations | | | | | | |
| 46 | P: Vaccination against diphtheria in the past 10 years | 0.88  (0.69-1.12) | 0.30 | 0.85  (0.66-1.1) | 0.21 | 0.03 |
| 47 | P: Vaccination against tetanus in the past 10 years | 0.77  (0.57-1.04) | 0.09 | 0.79  (0.58-1.07) | 0.12 | 0.02 |
| 48 | P: Vaccination against pertussis in the past 10 years | 0.89  (0.7-1.14) | 0.37 | 0.8  (0.61-1.05) | 0.11 | 0.02 |
| 49 | P: Vaccination against pneumococcus in the past 10 years | 0.61  (0.45-0.84) | <0.001 | 0.61  (0.43-0.86) | <0.001 | 0.004 |
| 50 | P: Vaccination against poliomyelitis in the past 10 years | 1.02  (0.81-1.3) | 0.84 | 1.01  (0.79-1.3) | 0.92 | 0.05 |
| 51 | P: Vaccination against hepatitis B in the past 10 years | 0.93  (0.74-1.18) | 0.57 | 0.82  (0.64-1.06) | 0.13 | 0.02 |
| 52 | A: Intended vaccination against diphtheria (in the future) | 0.96  (0.75-1.23) | 0.73 | 0.96  (0.74-1.26) | 0.78 | 0.05 |
| 53 | A: Intended vaccination against tetanus (in the future) | 1.11  (0.85-1.44) | 0.45 | 1.12  (0.85-1.47) | 0.41 | 0.04 |
| 54 | A: Intended vaccination against pertussis (in the future) | 0.78  (0.6-1.02) | 0.07 | 0.72  (0.53-0.96) | 0.03 | 0.01 |
| 55 | A: Intended vaccination against pneumococcus (in the future) | 0.68  (0.5-0.92) | 0.01 | 0.66  (0.48-0.9) | 0.01 | 0.007 |
| 56 | A: Intended vaccination against poliomyelitis (in the future) | 0.87  (0.68-1.13) | 0.30 | 0.87  (0.67-1.15) | 0.33 | 0.03 |
| 57 | A: Intended vaccination against hepatitis B (in the future) | 0.88  (0.68-1.14) | 0.33 | 0.82  (0.62-1.09) | 0.17 | 0.02 |
| 58 | P: Frequency of influenza vaccinations in the past 10 years | 0.73  (0.6-0.9) | <0.001 | 0.76  (0.62-0.94) | 0.01 | 0.007 |
| 59 | A: Intended vaccination against influenza (in the future) | 0.77  (0.6-0.98) | 0.03 | 0.81  (0.62-1.04) | 0.10 | 0.02 |
| 60 | K: Vaccination recommendation diphtheria | 1.73  (0.79-3.81) | 0.17 | 1.78  (0.78-4.05) | 0.17 | 0.02 |
| 60 | P: Vaccination against influenza H1N1 in the winter season 2009/2010 | 0.75  (0.55-1.03) | 0.08 | 0.79  (0.57-1.09) | 0.15 | 0.02 |
| 61 | K: Vaccination recommendation pertussis | 0.78  (0.58-1.07) | 0.12 | 0.79  (0.57-1.1) | 0.16 | 0.02 |
| 62 | K: Vaccination recommendation measles | 1.17  (0.86-1.59) | 0.31 | 1.12  (0.82-1.53) | 0.49 | 0.04 |
| 63 | K: Vaccination recommendation influenza | 0.71  (0.54-0.92) | 0.01 | 0.72  (0.54-0.96) | 0.02 | 0.01 |
| 64 | K: Vaccination recommendation pneumococcus | 1.14  (0.76-1.72) | 0.53 | 1.23  (0.81-1.88) | 0.34 | 0.03 |
| 65 | K: Vaccination recommendation poliomyelitis | 0.65  (0.43-0.97) | 0.04 | 0.72  (0.47-1.1) | 0.13 | 0.02 |
| 66 | K: Vaccination recommendation rabies | 1.15  (0.85-1.56) | 0.35 | 1.14  (0.83-1.56) | 0.42 | 0.04 |
| 68 | **A: Vaccinations are effective in preventing infectious diseases** | 0.69  (0.55-0.87) | <0.001 | **0.66**  **(0.52-0.83)** | **<0.001** | 0.003 |
| 69 | **A: Vaccinations are getting safer and more effective** | 0.65  (0.52-0.81) | <0.001 | **0.62**  **(0.5-0.78)** | **<0.001** | 0.002 |
| 70 | A: Immune system is weakened because of to many vaccinations | 1.08  (0.88-1.33) | 0.47 | 1.12  (0.91-1.39) | 0.28 | 0.03 |
| 71 | A: No vaccination because of the syringes | 0.78  (0.5-1.21) | 0.26 | 0.78  (0.5-1.23) | 0.29 | 0.03 |
| 72 | A: No vaccination because of the adverse effects | 1.05  (0.84-1.31) | 0.70 | 1.09  (0.87-1.37) | 0.46 | 0.04 |
| 73 | A: No vaccination because of the late effects | 0.99  (0.79-1.26) | 0.96 | 1  (0.78-1.27) | 0.97 | 0.05 |
| 74 | A: Compulsory vaccination for all adults | 0.87  (0.71-1.06) | 0.16 | 0.85  (0.69-1.04) | 0.12 | 0.02 |
| 75 | A: Compulsory vaccination for medical staff | 0.82  (0.67-1.01) | 0.07 | 0.79  (0.63-0.97) | 0.03 | 0.01 |
| 76 | A: Vaccinations in general | 0.73  (0.59-0.91) | <0.001 | 0.73  (0.59-0.91) | 0.01 | 0.005 |
| 77 | A: Vaccination against tetanus | 0.84  (0.62-1.13) | 0.24 | 0.85  (0.62-1.15) | 0.29 | 0.03 |
| 78 | A: Vaccination against influenza | 0.75  (0.61-0.92) | 0.01 | 0.76  (0.62-0.94) | 0.01 | 0.008 |
| 79 | K: Ever heard of human papillomavirus | 0.98  (0.79-1.22) | 0.88 | 0.87  (0.68-1.11) | 0.27 | 0.03 |
| Tick-borne infections | | | | | | |
| 80 | P: Frequency of private stays in the woods | 1.06  (0.87-1.29) | 0.58 | 1.14  (0.93-1.4) | 0.20 | 0.03 |
| 81 | K: Ticks transmit Borreliosis | 0.49  (0.26-0.93) | 0.03 | 0.51  (0.27-0.97) | 0.04 | 0.01 |
| 82 | K: Borreliosis is a serious disease | 0.71  (0.48-1.07) | 0.10 | 0.75  (0.49-1.15) | 0.18 | 0.02 |
| 83 | K: Children are particularly vulnerable to borreliosis | 1.26  (1.01-1.59) | 0.04 | 1.35  (1.06-1.7) | 0.01 | 0.008 |
| 84 | A: Worry to get infected with borreliosis | 1.04  (0.86-1.26) | 0.69 | 1.07  (0.87-1.3) | 0.53 | 0.04 |
| 85 | K: Ticks transmit tick-borne encephalitis (TBE) | 0.84  (0.54-1.29) | 0.42 | 0.89  (0.57-1.37) | 0.58 | 0.04 |
| 86 | K: TBE is a serious disease | 0.83  (0.56-1.24) | 0.37 | 0.93  (0.61-1.4) | 0.72 | 0.04 |
| 87 | K: Children are particularly vulnerable to TBE | 1.24  (0.97-1.59) | 0.09 | 1.28  (0.99-1.65) | 0.06 | 0.01 |
| 88 | A: Worry to get infected with TBE | 1.05  (0.85-1.29) | 0.65 | 1.11  (0.9-1.37) | 0.32 | 0.03 |
| 89 | **K: Avoidance of woods protects against tick bites** | 1.42  (1.16-1.74) | <0.001 | **1.37**  **(1.12-1.69)** | **<0.001** | 0.004 |
| 90 | K: Avoidance of meadows protects against tick bites | 1.43  (1.17-1.76) | <0.001 | 1.34  (1.09-1.65) | 0.01 | 0.005 |
| 91 | K: Long clothes protects against tick bites | 1.01  (0.82-1.25) | 0.92 | 1.03  (0.83-1.28) | 0.77 | 0.04 |
| 92 | K: Ankle-high shoes protects against tick bites | 1.08  (0.88-1.33) | 0.44 | 1.14  (0.92-1.41) | 0.22 | 0.03 |
| 93 | K: Wearing trousers in socks protects against tick bites | 1.09  (0.89-1.35) | 0.40 | 1.16  (0.93-1.45) | 0.18 | 0.02 |
| 94 | K: Anti-tick treatment protects against tick bites | 1.32  (1.05-1.65) | 0.02 | 1.29  (1.03-1.63) | 0.03 | 0.01 |
| 95 | K: Inspection of the body after stays in the woods protects against tick bites | 1.3  (1.03-1.64) | 0.03 | 1.33  (1.05-1.69) | 0.02 | 0.009 |
| 96 | K: After a tick bite: remove the tick immediately | 0.89  (0.65-1.22) | 0.47 | 0.97  (0.69-1.36) | 0.86 | 0.05 |
| 97 | K: After a tick bite: a doctor should remove the tick | 1.14  (0.93-1.38) | 0.20 | 1.21  (0.99-1.48) | 0.06 | 0.01 |
| 98 | K: After a tick bite: apply alcoholic solution on the tick before removing it | 1.4  (1.05-1.85) | 0.02 | 1.33  (1-1.77) | 0.05 | 0.01 |
| 99 | K: After a tick bite: apply toothpaste on the tick before removing it | 1.1  (0.74-1.64) | 0.63 | 1.07  (0.71-1.6) | 0.75 | 0.04 |
| 100 | K: After a tick bite: apply oil on the tick before removing it | 1.02  (0.72-1.42) | 0.93 | 0.99  (0.7-1.4) | 0.97 | 0.05 |
| 101 | K: After a tick bite: remove the tick by pulling it straight | 0.91  (0.73-1.13) | 0.40 | 0.9  (0.72-1.12) | 0.33 | 0.03 |
| 102 | K: After a tick bite: unscrew the tick | 1.19  (0.97-1.47) | 0.10 | 1.25  (1.01-1.55) | 0.04 | 0.01 |
| 103 | P: Implementation of avoidance of woods | 1.22  (0.96-1.55) | 0.10 | 1.22  (0.96-1.56) | 0.11 | 0.02 |
| 104 | P: Implementation of avoidance of meadows | 1.25  (1-1.57) | 0.05 | 1.27  (1.01-1.59) | 0.04 | 0.01 |
| 105 | P: Implementation of long clothes | 0.83  (0.68-1.01) | 0.06 | 0.89  (0.73-1.08) | 0.24 | 0.03 |
| 106 | P: Implementation of ankle-high shoes | 0.92  (0.76-1.12) | 0.42 | 0.98  (0.8-1.19) | 0.80 | 0.05 |
| 107 | P: Implementation of wearing trousers in socks | 0.74  (0.61-0.91) | <0.001 | 0.78  (0.63-0.96) | 0.02 | 0.009 |
| 108 | **P: Implementation of anti-tick treatment** | 0.73  (0.6-0.9) | <0.001 | **0.71**  **(0.57-0.88)** | **<0.001** | 0.003 |
| 109 | P: Implementation of inspection of the body after stays in the woods | 0.79  (0.65-0.97) | 0.02 | 0.79  (0.64-0.97) | 0.02 | 0.01 |
| 110 | P: Implementation of removing the tick immediately | 0.77  (0.6-1) | 0.05 | 0.78  (0.6-1.02) | 0.07 | 0.02 |
| 111 | P: Implementation of removing the tick at a doctor's office | 0.96  (0.78-1.18) | 0.70 | 1.02  (0.82-1.26) | 0.86 | 0.05 |
| 112 | P: Implementation of applying alcoholic solution on the tick before removing it | 1.1  (0.87-1.39) | 0.43 | 1.09  (0.86-1.38) | 0.48 | 0.04 |
| 113 | P: Implementation of applying toothpaste on the tick before removing it | 0.8  (0.62-1.04) | 0.09 | 0.82  (0.63-1.07) | 0.14 | 0.02 |
| 114 | P: Implementation of applying oil on the tick before removing it | 0.84  (0.66-1.08) | 0.17 | 0.86  (0.67-1.11) | 0.26 | 0.03 |
| 115 | P: Implementation of removing the tick by pulling it straight | 0.9  (0.74-1.11) | 0.32 | 0.88  (0.72-1.09) | 0.23 | 0.03 |
| 116 | P: Implementation of unscrewing the tick | 1.13  (0.92-1.37) | 0.24 | 1.19  (0.97-1.46) | 0.10 | 0.02 |
| Antibiotics | | | | | | |
| 117 | K: Antibiotics are effective against bacteria | 0.88  (0.65-1.2) | 0.43 | 0.83  (0.6-1.14) | 0.19 | 0.03 |
| 118 | K: Antibiotics are effective against viruses | 0.93  (0.73-1.18) | 0.55 | 0.98  (0.76-1.25) | 0.48 | 0.05 |
| 119 | K: Penicillin is an antibiotic | 1.11  (0.8-1.54) | 0.55 | 1.11  (0.79-1.56) | 0.88 | 0.04 |
| 120 | K: Paracetamol is an antibiotic | 0.96  (0.63-1.47) | 0.86 | 1.03  (0.66-1.59) | 0.87 | 0.05 |
| 121 | K: Ibuprofen is an antibiotic | 0.85  (0.57-1.28) | 0.44 | 0.9  (0.59-1.37) | 0.72 | 0.04 |
| 122 | K: If an antibiotic is not taken as prescribed, then germs become resistant | 1.11  (0.87-1.4) | 0.40 | 1.13  (0.88-1.44) | 0.66 | 0.03 |
| 123 | K: If someone takes often antibiotics, then her/his body becomes immune | 0.88  (0.7-1.11) | 0.29 | 0.9  (0.71-1.14) | 0.62 | 0.04 |
| 124 | P: Asked GP for antibiotics because of a cold | 1.05  (0.77-1.42) | 0.77 | 1.1  (0.81-1.5) | 0.88 | 0.04 |
| 125 | P: Antibiotics in stock at home | 1.05  (0.66-1.68) | 0.84 | 1.09  (0.68-1.77) | 0.70 | 0.04 |
| 126 | P: Taking antibiotics according to recommendation | 0.72  (0.49-1.07) | 0.11 | 0.75  (0.5-1.12) | 0.22 | 0.02 |
| 127 | P: Stop antibiotic therapy when feeling better | 1.07  (0.79-1.45) | 0.65 | 1.06  (0.77-1.45) | 0.61 | 0.04 |
| 128 | P: Share the antibiotics with relatives | 0.88  (0.51-1.55) | 0.66 | 0.92  (0.52-1.64) | 0.69 | 0.05 |
| 129 | P: No intake of antibiotics at all | 1.1  (0.89-1.37) | 0.38 | 1.08  (0.86-1.35) | 0.51 | 0.04 |
| 130 | A: Worry about antibiotic resistances | 1.03  (0.83-1.27) | 0.80 | 1.08  (0.87-1.35) | 0.66 | 0.04 |
| 131 | P: Last time taking antibiotics:  asked for the antibiotic | 1.23  (0.9-1.67) | 0.19 | 1.28  (0.93-1.76) | 0.20 | 0.02 |
| 132 | P: Last time taking antibiotics:  according to recommended number of pills | 0.68  (0.42-1.11) | 0.12 | 0.61  (0.37-1.03) | 0.16 | 0.01 |
| 133 | P: Last time taking antibiotics:  according to recommended time interval of intake | 0.92  (0.67-1.27) | 0.63 | 0.96  (0.7-1.34) | 0.87 | 0.05 |
| 134 | P: Last time taking antibiotics:  according to recommended duration of antibiotic intake | 1.08  (0.74-1.57) | 0.70 | 1.02  (0.69-1.51) | 0.97 | 0.05 |

^a^Wald test with the null hypothesis that the respective OR is equal to one.

^b^Adjusted for age at baseline (fitted as fractional polynomial), sex, and highest completed education

Bold: items with adjusted odds ratios significantly different from one after controlling the FDR

A: Question about attitudes

ARI: Acute respiratory infection

FDR: False discovery rate

FREQ: Question about frequency of infections

GP: General practitioner

K: Question about knowledge

ON: Online questionnaire

P: Question about practice

X: Question about well-being
